# Electrochemical Detection of Endosulfan Using an AONP-PANI-SWCNT Modified Glassy Carbon Electrode

**DOI:** 10.3390/ma14040723

**Published:** 2021-02-04

**Authors:** Kgotla K. Masibi, Omolola E. Fayemi, Abolanle S. Adekunle, Amal M. Al-Mohaimeed, Asmaa M. Fahim, Bhekie B. Mamba, Eno E. Ebenso

**Affiliations:** 1 Department of Chemistry, School of Physical and Chemical Sciences, Faculty of Natural and Agricultural Sciences, North-West University (Mafikeng Campus), Private Bag X2046, Mmabatho 2735, South Africa; masibikk@gmail.com (K.K.M.); Omolola.Fayemi@nwu.ac.za (O.E.F.); 2 Material Science Innovation and Modelling (MaSIM) Research Focus Area, Faculty of Natural and Agricultural Sciences, North-West University (Mafikeng Campus), Private Bag X2046, Mmabatho 2735, South Africa; 3 Department of Chemistry, Obafemi Awolowo University, Ile-Ife 220005, Nigeria; aadekunle@oauife.edu.ng; 4 Department of Chemistry, College of Science, King Saud University, P.O. Box 22452, Riyadh 11495, Saudi Arabia; muhemeed@ksu.edu.sa; 5 Green Chemistry Department, National Research Centre, Dol, Cairo 12622, Egypt; asmaamahmoud8521@gmail.com; 6 Institute of Nanotechnology and Water Sustainability, College of Science, Engineering and Technology, University of South Africa, Johannesburg 1709, South Africa; mambabb@unisa.ac.za

**Keywords:** electrochemical sensor, metal-oxide nanoparticles, voltammetric techniques, endosulfan, nanocomposite

## Abstract

This report narrates the successful application of a fabricated novel sensor for the trace detection of endosulfan (EDS). The sensor was made by modifying a glassy-carbon electrode (GCE) with polyaniline (PANI), chemically synthesized antimony oxide nanoparticles (AONPs), acid-functionalized, single-walled carbon nanotubes (fSWCNTs), and finally, the AONP-PANI-SWCNT nanocomposite. The electrochemical properties of the modified electrodes regarding endosulfan detection were investigated via cyclic voltammetry (CV) and square-wave voltammetry. The current response of the electrodes to EDS followed the trend GCE-AONP-PANI-SWCNT (−510 µA) > GCE-PANI (−59 µA) > GCE-AONPs (−11.4 µA) > GCE (−5.52 µA) > GCE-fSWCNTs (−0.168 µA). The obtained results indicated that the current response obtained at the AONP-PANI-SWCNT/GCE was higher with relatively low overpotential compared to those from the other electrodes investigated. This demonstrated the superiority of the AONP-PANI-SWCNT-modified GCE. The AONP-PANI-SWCNT/GCE demonstrated good electrocatalytic activities for the electrochemical reduction of EDS. The results obtained in this study are comparable with those in other reports. The sensitivity, limit of detection (LoD), and limit of quantification (LoQ) of AONP-PANI-SWCNT/GCE towards EDS was estimated to be 0.0623 µA/µM, 6.8 µM, and 20.6 µM, respectively. Selectivity, as well as the practical application of the fabricated sensor, were explored, and the results indicated that the EDS-reduction current was reduced by only 2.0% when interfering species were present, whilst average recoveries of EDS in real samples were above 97%.

## 1. Introduction

Endosulfan (EDS) is a broad-spectrum organochlorine pesticide (OCP) primarily used on a variety of cereals, fruits, vegetables, and cotton globally [1,2,3]. It has the molecular formula C_9_H_6_C_l6_O_3_S and molecular weight of 406.93 g/mol, with a melting point ranging from 208 °C to 210 °C. Commercially, EDS is available as a combination of its isomers, α- and β-endosulfan, in the ratio 7:3 [1,4]. Similar to persistent organochlorine pesticides (POPs), OCPs such as EDS tend to resist biological degradation and are prone to travel over long distances in air before being redeposited, even to areas where they were never used [1,5,6]. Despite the ban on most of these chemicals decades ago, OCPs are amongst the most commonly detected analytes globally, and they continue to be found in several matrices such as soil, river sediments, and surface water. Some developing countries are dependent on OCPs in large quantities for agricultural purposes due to their cost efficiency and pest-control efficacy [7,8]. EDS is reportedly one of the most abundant OCPs in the global atmosphere, and, owing to its stability as well as persistence, it has found an entry into the food chain and, consequently, to the human system, where it is stored in the lipid portion of adipose tissue [7,9,10,11].

Human reactions to low-level EDS exposure include convulsions, psychiatric disturbances, epilepsy, paralysis, impaired memory, immunosuppression, birth defects, neurological disorders, and even death [3,12,13,14]. Neurotoxicity is the major endpoint of concern in both experimental animals and human beings. Subacute as well as chronic toxicity studies of EDS in animals suggest that the testes, kidneys, liver, as well as the entire immune system are the main targets [3,13]. In general, analyses of endosulfan have been achieved mainly by conventional analytical methods such as ultraviolet-visible (UV-Vis) spectroscopy [15], gas chromatography (GC) coupled with various detectors including the electron-capture detector (ECD) [9,16,17,18], flame-photometric detector (FPD) [19], mass spectrometry (MS) [20], and high-performance liquid chromatography (HPLC) [21,22,23]. Each of these methods, as much as they provide significant sensitivity and reliability, has a number of drawbacks. One of these drawbacks is that they require laborious steps of sample extraction and cleanup, resulting in time delays [8,24]. Therefore, there is a need for the development of novel methods that are cost efficient, simple, more sensitive, accurate, and suitable for onsite analysis of EDS and other important environmental analytes.

In recent times there has been great progress in nanotechnology. A variety of nanomaterials such as graphene nanofibers, metal nanoparticles (NPs), carbon nanotubes, polymers, and metal-oxide NPs with distinctive chemical and physical properties have been developed and incorporated in electrochemical sensors for the detection of different OCPs with outstanding results [25]. To the best of our knowledge, there are only a few reports on the electrochemical detection of EDS [8,26,27,28,29,30]. Ribeiro et al. [8] described an electroanalytical methodology for the detection of EDS on a hanging mercury-drop electrode (HMDE). Prabu and Manisankar [26] employed a square wave voltammetry method for the electrochemical studies of EDS on a glassy carbon electrode. Manisankar et al. [27] electropolymerized polypyrrole on the surface of GCE for the electrochemical detection of EDS using voltammetry methods. Priyantha and Thirana [28] coated the surface of GCE with a thin film of 5,10,15,20-tetraphenylporphyrinatoiron (III) chloride (Fe(III)TPPCl) for the voltammetric determination of EDS. In the same vein, a nonenzymatic electrochemical sensor with a copper-oxide-modified gold electrode (CuO/Au) was developed to detect α-endosulfan in water samples using Differential Pulse Voltammetry (DPV) and amperometry techniques in this study. The sensor showed a linear range of 4–20 nM, with a limit of detection (LoD) of 8.3093 µA and a sensitivity of 0.03 µA nM-1 [29]. Baybars et al. [30] developed a novel asymmetric zinc (II) phthalocyanines (ZnPc), including three boron dipyrromethenes (BODIPY) and a single-walled carbon nanotube (SWCNT) BODIPY-Phthalocyanine-SWCNT Hybrid Platform-modified GCE electrode, for the detection of pesticides such as methyl parathion, deltamethrin, chlorpyrifos, and spinosad in juice samples. The electrochemical behavior of the nonenzymatic electrode (GCE/SWCNT-ZnPc) was determined using cyclic voltammetry and differential pulse voltammetry. The sensor demonstrated high selectivity for methyl parathion with a low limit of detection of 1.49 nM.

In this study, a novel electrochemical sensor which is highly selective and sensitive to EDS was developed by modifying a glassy-carbon electrode (GCE) with a nanocomposite of polyaniline (PANI) and antimony oxide nanoparticles (AONPs), supported on functionalized single-walled carbon nanotubes (PANI-AONPs-fSWCNTs). AONPs are unique among all other metal oxides, starting from group V to group VI of the periodic table [31,32,33]. AONPs have higher abrasion resistance, refractive index, absorbability, proton conductivity, and outstanding mechanical strength compared to bulk Sb_2_O_3_ [31,34,35]. The unique properties of AONPs have been explored in many different fields of application, including catalysis, fire retardants, glasses, and sensors [31,32,33,34,35]. Single-walled carbon nanotubes are an ideal support material for metal and metal-oxide nanoparticles given their low reactivity to sp2-hybridized carbon atoms, their high surface area, tensile mechanical strength, good electrical conductivity, as well as the fact that all of the carbon atoms in SWCNTs are surface atoms [36,37,38]. The use of SWCNTs as an electrode-supporting material for electrochemical detection of some important analytes has previously been reported. Wei et al. [38] fabricated an electrochemical sensor for the detection of Hg (II) in water, based on a gold electrode modified with thiophenol-functionalized SWCNTs. In this paper, we therefore report the successful application of a fabricated novel sensor for the trace detection of endosulfan (EDS). The sensor was made by modifying a glassy-carbon electrode (GCE) with the nanocomposite PANI-AONPs-fSWCNTs. The electrochemical properties of the modified electrode regarding endosulfan detection were investigated via cyclic voltammetry (CV) and square-wave voltammetry. Selectivity, as well as practical application of the fabricated sensor, were explored.

## 2. Experimental

### 2.1. Materials and Reagents

A glassy-carbon electrode, 3 mm in diameter, made from silver-silver chloride (Ag|AgCl) in saturated KCl, which was used as a reference electrode, and the counter electrode (platinum disk) were purchased from CH Instruments Inc., Austin, TX, USA. The GCE polishing pads used alongside the alumina micro powder (1.0, 0.3, and 0.05 μm alumina slurries) were obtained from Buehler, Lake Bluff, IL, USA. Pristine SWCNTs (90% purity, 0.7–1.1 nm), endosulfan (C_9_H_6_Cl_6_O_3_S), N,N-Dimethylformamide (DMF), ammonium peroxydisulfate (APS) ((NH_4_)_2_S_2_O_8_), nitric acid (HNO_3_), methanol, polyvinyl alcohol (PVA), antimony chloride (SbCl_3_), sodium hydroxide (NaOH), potassium phosphate (Na_2_HPO_4_), hydrochloric acid (HCl), aniline (99%), sodium phosphate (NaH_2_PO_4_), and ethanol were obtained from Sigma-Aldrich (St. Louis, MO, USA) and Merck chemicals (Darmstadt, Germany). They were all of analytical grade. Solutions were prepared using ultrapure water with a resistivity of 18.2 MΩcm, which was obtained from a Milli-Q Water System (Millipore Corp., Bedford, MA, USA). The NaH_2_PO_4_ and Na_2_HPO_4_ solutions adjusted with NaOH were used for the preparation of 0.1 M phosphate buffer solution (PBS) of pH 7.0. All solutions were de-aerated by bubbling nitrogen prior to each electrochemical experiment.

### 2.2. Preparation of AONPs, PANI and fSWCNTs

Information on the preparation and characterization of AONPs, PANI, fSWCNTs has been reported in our previous studies [39].

### 2.3. Preparation of Nanocomposites and Electrode Modification

About 2 mg of fSWCNT suspension in 2 mL DMF was doped with 2 mg of both AONPs and PANI. The resultant mixture was stirred at room temperature for 48 h to obtain a putty form of AONP-PANI-SWCNTs composite. This nanocomposite was dried overnight at 25 °C in order to evaporate the solvent [39]. The drop-cast method was used for the modification of the electrode. The surface of GCE was gently polished with 0.3 µm alumina slurries dispensed on a polishing pad prior to rinsing with distilled water. The GCE-working electrode was then sonicated in ethanol for 5 min and in distilled water again for 5 min to remove any particles that may have been trapped on the surface. Then, 5 mg of each of the prepared nanomaterials, i.e., fSWCNTs, PANI, AONPs, and AONP-PANI-SWCNT nanocomposite, was suspended in 1 mL DMF. A suspension of each of these materials was then dispersed by ultrasonic vibration for 30 min. Following this, a 20-µL aliquot of each of these dispersions was dropped onto the surface of the polished GCE and dried at 50 °C to obtain the modified electrodes used for the analytical studies [40,41].

### 2.4. Electrochemical Study

All electrochemical experiments were done with GPES software version 4.9 running on Autolab Potentiostat PGSTAT 302 (Eco Chemie, Utrecht, The Netherlands). The electrochemical setup consisted of a three-electrode system, with GCE as the working electrode, platinum (Pt) wire as the counter electrode, and Ag|AgCl in saturated KCl as the reference electrode.

## 3. Results and Discussion

### 3.1. Electrocatalytic Reduction of Endosulfan

**Figure 1:**
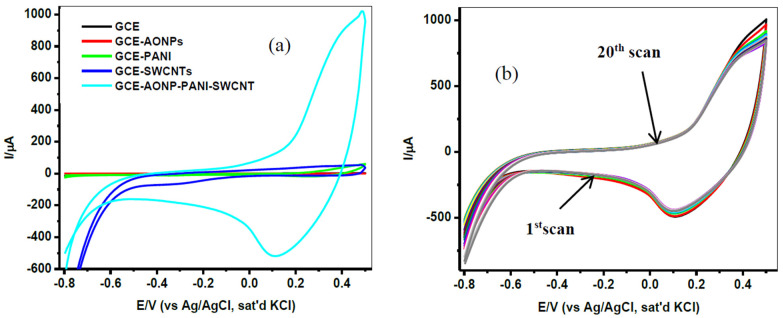
(**a**) Cyclic voltammograms of modified electrodes in 86 μM EDS prepared in 2:1 water/acetonitrile solution containing 0.1 M H_2_SO_4_ at a scan rate of 25 mVs^−1^. (**b**) Cyclic voltammograms (20 scans) of AONP-PANI-SWCNT-modified GCE in 86 μM EDS prepared in 2:1 water/acetonitrile solution containing 0.1 M H_2_SO_4_ at a scan rate of 25 mVs^−1^.

The electrochemical behavior of modified electrodes was investigated via cyclic voltammetry (CV) in 86 μM EDS made in 2:1 water/acetonitrile containing 0.1 M H_2_SO_4_ within a potential window of −0.8 to 0.5 V (vs Ag/AgCl satd’d KCl) at scan rate of 25 mVs^−1^. The cyclic voltammograms (not shown) demonstrated well-defined, irreversible EDS-reduction peaks at −0.45 V (bare GCE), −0.27 V (GCE-fSWCNTs), −0.37 V (GCE-AONPs), −0.31 V (GCE-PANI), and 0.13 V for the GCE-AONP-PANI-SWCNT electrode. This simply suggests that EDS had a different reduction potential at each electrode studied. Comparatively, the reduction current was different for each electrode, with the GCE-AONP-PANI-SWCNT electrode having a higher reduction current (−510 µA) compared to the bare electrode (−5.52 µA) and other modified electrodes investigated (Figure 1a). In fact, the EDS-reduction current at the AONP-PANI-SWCNT electrode was approximately 92, 3000, 45, and 9 times higher than those of the bare GCE, GCE-fSWCNTs, GCE-AONPs, and GCE-PANI electrodes, respectively. As observed, the highest reduction current was recorded at the AONP-PANI-SWCNT-modified GCE, which suggests enhanced electrocatalysis of the modified electrode toward EDS. Similar studies on enhanced EDS current at chemically-modified electrodes have been reported [8]. Since the AONP-PANI-SWCNT-modified GCE performed best, its stability and resistance to fouling due to the product of EDS reduction were further explored using CV experiments (20 scans) in 86 μM EDS, prepared in 2:1 water/acetonitrile containing 0.1M H_2_SO_4_, at a scan rate of 25 mVs^−1^ over a potential window of −0.8–0.5 V (Figure 1b). The results showed the EDS-reduction current to be stable with a very insignificant current drop between the first and the twentieth scan (Figure 1b). Thus, the AONP-PANI-SWCNT-modified electrode evidently demonstrated significant resistance to the electrode-fouling effect. This result therefore suggests that the fabricated sensor is sustainable and can be routinely used for the detection and quantification of endosulfan pesticides in polluted water.

### 3.2. Effect of Scan Rate on Endosulfan Reduction

**Figure 2:**
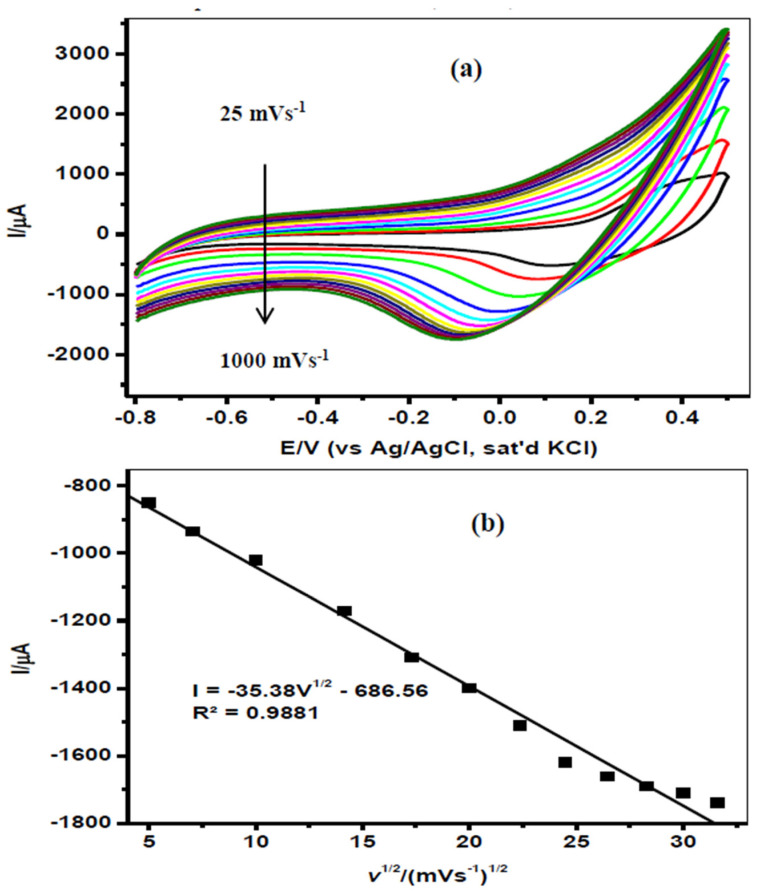
(**a**) Cyclic voltammograms of AONPs-PANI-SWCNT-modified GCE in 86 μM EDS prepared in 2:1 water/acetonitrile containing 0.1M H_2_SO_4_ at a scan rate of 25–1000 mVs^−1^. (**b**) Plot of the anodic peak current (I_pa_) versus the square root of the scan rate (*v*) for AONP-PANI-SWCNT-modified GCE in EDS.

The effect of the scan rate (range 25–1000 mVs^−1^) was investigated by conducting CV experiments using AONP-PANI-SWCNT-modified GCE in 86 μM EDS prepared in 2:1 water/acetonitrile containing 0.1 M H_2_SO_4_ (Figure 2a). It was observed that the EDS reduction peak current increased with the increasing scan rate (Figure 2b). The plot of the cathodic peak current (I_pa_) versus the square root of scan rate (*v*^1/2^) was linear, suggesting a diffusion-controlled reaction at the electrode surface [40,42,43]. The linear graph is defined by the equation I = −35.38*v*^1/2^ − 686.56 (R^2^ = 0.9881). Previous reports have suggested that EDS cannot dissociate due to its molecular structure [44]. It has been further submitted that EDS has no oxidizing properties, no acid proton, and no reasonable amount of basic centers. However, EDS is said to be sensitive to acids, alkalis, and moisture; thus, its electrochemical reduction is heavily dependent on the cell constituents, supporting electrolyte, and potential window [28,44]. The structure of EDS has numerous chlorine atoms located at different positions; thus, it is prone to reduction at these carbon-chlorine positions. However, reports have suggested that a transition state exists for the transfer of electrons in the carbon-chlorine bond that is oriented perpendicular to the surface of the electrode with the chlorine atom nearest to the electrode. The reduction occurs at the C(7)-Cl bond, and the structure resulting from this bond rupture is comparatively the most stable. This is supported by the evaluation of the lowest unoccupied molecular orbital (LUMO) of EDS [8].

**Scheme 1:**
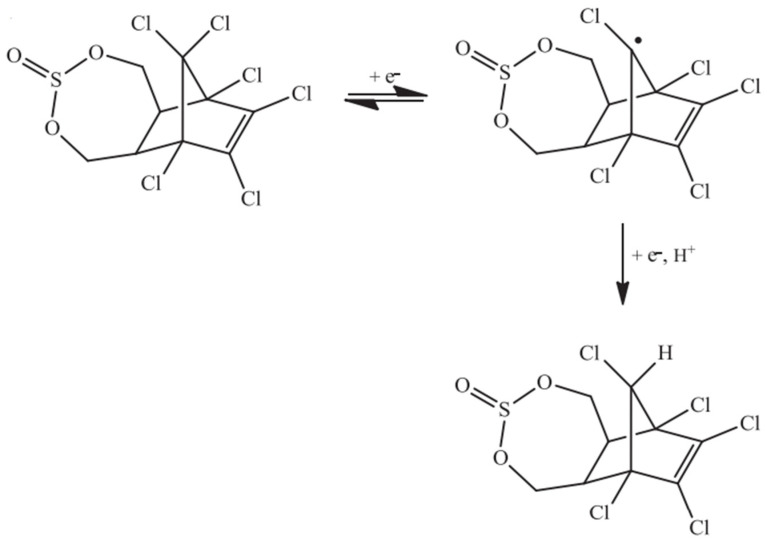
EDS-reduction mechanism [8].

It was deduced that the most feasible mechanism of EDS reduction in an acidic medium involves two steps. The first step is a radical formation as a result of the reduction of the chlorine atom connected to the carbon in the C(7) position, and this involves only one electron. The radical formation is reduced on the second step involving one more proton and electron [8]. The proposed mechanism is shown in Scheme 1.

### 3.3. Electroanalysis of Endosulfan

**Figure 3:**
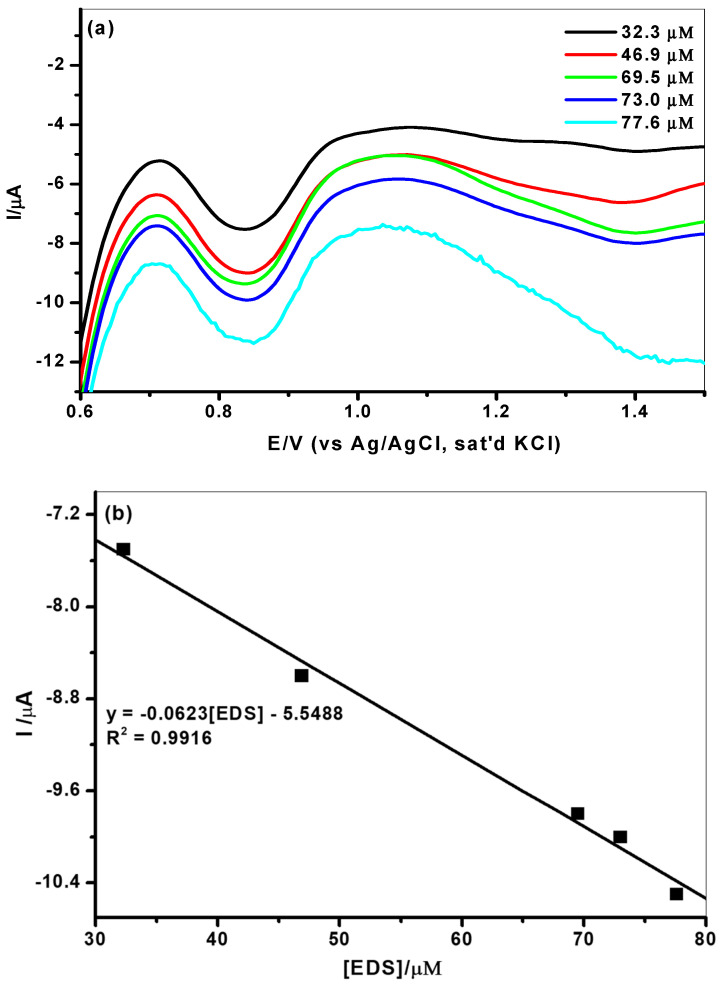
(**a**) Square-wave voltammograms of AONP-PANI-SWCNT-modified GCE in 32.3 µM to 77.6 µM EDS prepared in 2:1 water/acetonitrile containing 0.1 M H_2_SO_4_. (**b**) The plot of current response versus EDS concentration.

The effect of current response of varying EDS concentrations (32.3–77.6 µM) prepared in 2:1 water/acetonitrile containing 0.1 M H_2_SO_4_ on AONP-PANI-SWCNT-modified GCE was measured by square-wave voltammetry (SWV) (Figure 3a). In Figure 3a, it can be seen that the EDS reduction peak current increased with a climbing concentration of EDS, whilst there was also a gradual negative shift in potential. Figure 3b depicts a plot of current response versus EDS concentration. The plot yielded a linear relationship with the equation I = −0.0623 (EDS) − 5.548 (R^2^ = 0.9916). The limit of detection (LoD) and limit of quantification (LoQ) for the electrodes represent the lowest analyte concentration in a sample that can be detected or quantified with acceptable accuracy and precision. They were determined using the equations LoD = 3.3 δ/m, where δ is the relative standard deviation of the intercept of the y-coordinates and m is the slope of the graph [45], and LoQ = 10 δ/m with the data from Figure 3a. The LoD, LoQ, and sensitivity of the AONP-PANI-SWCNT-modified GCE regarding EDS detection were calculated to be 6.8 µM, 20.6 µM, and 0.2086 µA/µM, respectively. The LoD obtained in this study (5.22 µM) was found to be lower than the 20 µM reported at the molecular-imprinted polymer (MIP) [45] but higher than the 0.297 µM reported at the hanging mercury-drop electrode (HMDE) [8]. However, the resistance to the electrode fouling effect and enhanced stability demonstrated by the electrode compared to those in similar studies cannot be overemphasized. It is important to mention that there is a paucity of literature on electrochemical detection of EDS, and therefore, this study presents significant scientific contributions using AONP-PANI-SWCNT-composite-modified GCE for EDS detection and quantification. Table 1 summarizes the analytical performance of AONP-PANI-SWCNT-modified GCE compared with other sensors reported for EDS detection.

The re-usability of the AONP-PANI-SWCNT sensor was tested several times in the EDS solution. Initially, the current dropped minimally between the first and the third runs. This might have been due to EDS adsorption or presence of its oxidation intermediated on the sensor surface. However, the sensor was renewed in a freshly prepared electrolyte solution, and the analysis was repeated, leading to about 80% of the initial EDS current at the electrode surface. The results therefore suggest that the electrode can be reused after an experiment once the surface is renewed. The renewed electrode was also stored in a refrigerator for two weeks and used again with no significant loss in the observed EDS-current response.

### 3.4. Endosulfan Interference Studies

**Figure 4:**
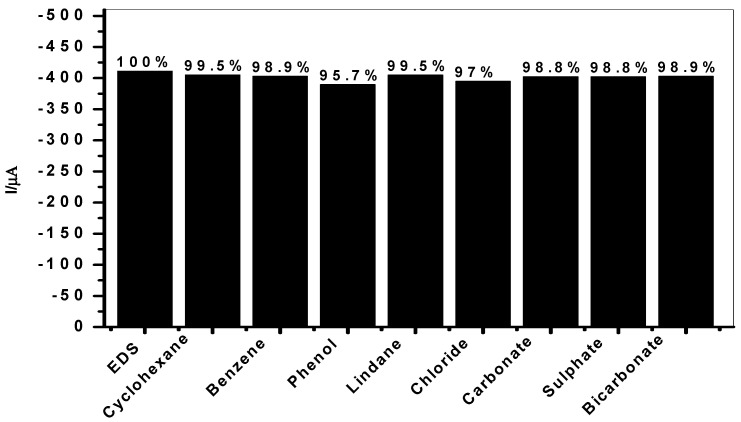
EDS-reduction current response to AONP-PANI-SWCNT-modified GCE as a percentage in the presence and absence of the selected interfering species by CV experiment.

Interference studies were done in order to investigate the selectivity of the modified electrode regarding EDS determination with the CV technique. Possible interfering species selected for the study included some electroactive organic compounds such as benzene (C_6_H_6_), cyclohexane (C_6_H_12_), phenol (C_6_H_5_OH), and organochlorine pesticide, e.g., lindane (C_6_H_6_Cl_6_) and various anions such as chloride (Cl^−^), carbonate (CO_3_^2−^), sulfate (SO_4_^2−^), and bicarbonate (HCO_3_^−^). These were selected based on their solubility in water and soil, structural similarity, as well as the fact that these species may be found in soil or water from industrial areas and agricultural land. Figure 4 shows the variation of EDS-reduction current as a percentage in the presence of interfering species in AONP-PANI-SWCNT-modified GCE at a scan rate of 25 mVs^−1^ using the cyclic voltammetry method. The results indicate that the EDS-reduction signal in the presence of the interfering species had an insignificant current drop of approximately 2.0% in all cases, confirming the outstanding selectivity of the proposed sensors regarding EDS detection.

### 3.5. Real Sample Analysis

In order to determine the prospects for a practical application of the proposed sensor, SWV experiments were conducted utilizing river and tap-water samples. Samples of river water were obtained from Crocodile River in the North West province of South Africa, and the tap-water samples were obtained from the local municipal water supply. The GPS coordinates of the sample collection points are noted below. The collected water samples were used without further purification, and EDS standards with concentrations of approximately 20 µM–196 µM were prepared in a 2:1 sample water/acetonitrile containing 0.1 M H_2_SO_4_. SWV experiments were conducted to obtain calibration curves, as well as to determine the concentration of EDS in the samples. The obtained results are presented in Table 2. The results obtained for the proposed sensor indicated significant recoveries of EDS with a 99.4% and 97.5% average for river water and tap water, respectively.

## 4. Conclusions

In this paper, we report on the development of modified GCE sensors for the electrochemical detection of endosulfan (EDS). Electrocatalytic reduction of endosulfan was successfully done via CV technique, and the AONP-PANI-SWCNT-modified GCE yielded the highest current response. A stability study showed that the AONP-PANI-SWCNT-modified GCE demonstrated little or no current drop (after 20 scans) in EDS, suggesting its high resistance to electrode-fouling effects in the analyte. This result therefore implies that the fabricated sensor is sustainable and can be used routinely for the detection and quantification of endosulfan in pesticide-polluted water, soil, and sediments. Through CV experimentation, the effect of the scan rate was investigated, and the plot of the reduction-peak currents against the square root of the scan rate showed a linear relationship, suggesting a diffusion-controlled electrode process. The LoD of the AONP-PANI-SWCNT-modified GCE regarding endosulfan compared favorably and was even better than that of some other sensors reported in previous studies. The proposed sensor further demonstrated good anti-interference behavior and selectivity regarding the detection of endosulfan in the presence of interferents. A real-sample analysis of endosulfan in river and tap water with satisfactory percentage recoveries demonstrated the potential of the developed sensor for analytical application.

## Figures and Tables

**Table 1:** Analytical performance of the proposed sensor for electrochemical detection of EDS.

LoD Values Compared with Other Studies				
Composite Electrode	LoD (µM)	Linear Range (µM)	Sensitivity (µA µM^−1^)	Reference
MIP	20	20–120	-	[46]
HMDE	0.297	0.154–0.157	0.0188	[8]
C18/CPE	9.83 × 10^−6^	-	-	[47]
GCE-Ph-NH_2_/SWCNT/PEG/FDMA/endosulfan hapten/antiendosulfan IgG	0.025	0.025–49.2	-	[2]
GCE-AONPs-PANI-SWCNT	6.8	32.3–77.6	0.0623	Present study

Abbreviations: MIP—Molecularly-imprinted polymer, HMDE—Hanging mercury-drop electrode, C18—Silicon network-modified with chains of 18 atoms of carbon in its surface, CPE—Carbon-paste electrode, GCE—Glassy-carbon electrode, Ph-NH_2_—4–aminophenyl/phenyl, SWCNT—Single-walled carbon nanotube, PEG—Poly(ethylene glycol), FDMA—Ferrocenedimethylamine, AONPs—Antimony-oxide nanoparticles, PANI—Polyaniline.

**Table 2:** Determination of the concentration of EDS in river water and tap-water samples by SWV. (^a^ Average value of three determinations).

Sample	Compound	Added (µM)	Found ^a^ (µM)	Recovery (%)	RSD (%)
River water	EDS	20	19.1	95.5	1.23
		40	40.2	100.5	1.08
		80	83.3	104	2.18
		100	97.5	97.5	1.06
Tap water	EDS	46	42.7	92.9	1.87
		96	96.1	100.1	4.07
		146	145.9	99.9	1.19
		196	190.0	96.9	2.52

## Data Availability

The data that support the findings of this study are available from the corresponding author upon reasonable request.
